# Teaching Case 5-2018: Integrated morphological and immunological work-up of neurosurgical specimen allows accurate diagnosis of neuroinflammatory lesions: an example of acute disseminated encephalomyelitis (ADEM) associated with anti-MOG antibodies 

**DOI:** 10.5414/NP301136

**Published:** 2018-08-13

**Authors:** Julia Lang, Ariane Biebl, Andreas Gruber, Beate Maier-Hiebl, Johannes A. Hainfellner, Romana Höftberger, Ellen Gelpi

**Affiliations:** 1 Institute of Neurology, Medical University of Vienna, Austria,; 2 Department of Pediatrics,; 3 Department of Pediatric Radiology,; 4 Department of Neurosurgery, Kepler University Hospital, Linz, Austria, and; 5 Neurological Tissue Bank of the Biobanc-Hospital Clinic-IDIBAPS, Barcelona, Spain

**Keywords:** acute disseminated encephalomyelitis, ADEM, MOG antibodies, autoimmune encephalitis, demyelination

## Abstract

No Abstract available.

We present histopathological and immunological findings of a 4-year-old male patient. He presented with fatigue, gait disturbances, and cognitive alterations. MRI revealed diffuse bilateral FLAIR, and T2-hyperintense and inhomogeneous signal alteration in thalamus and midbrain, with additional involvement of hypothalamus, capsula interna, and segments of globus pallidus, as well as of parietal white matter. A midline glioma was initially suspected. After stereotactic biopsy, histological examination revealed small fragments of CNS tissue with diffuse reactive astrogliosis. Remarkably, perivascular areas of inflammation and demyelination with loss of myelin basic protein (MBP) and myelin oligodendrocyte glycoprotein (MOG) immunoreactivity, preserved TPPP-positive oligodendrocytes, and preserved axonal profiles were observed (Figure 1A, B,C, D, E, F). In these areas, perivascular lymphocytic cuffs consisted predominantly of CD4-positive T-cells and CD20- and CD79A-positive B-cells and less perivascular and parenchymal CD3- and CD8-positive T-cells. In addition, abundant activated HLA-DR and CD68-positive microglia/macrophages (Figure 1E) containing myelin degradation products were apparent. There was also mild complement deposition around the vessels (Figure 1F). Immunohistochemical testing for viral antigens (*Herpes simplex* virus 1 and 2, *Varicella zoster*, CMV, FSME, and measles) was negative. These morphological features were suggestive of acute disseminated encephalomyelitis (ADEM). To further evaluate a possible autoimmune origin of the lesion, serum of the patient was tested for the presence of autoantibodies in a cell-based assay and revealed anti-MOG antibodies (Figure 1G, H, I), thus confirming the diagnosis of ADEM associated with anti-MOG antibodies. In our case, the integrated histopathological and serological investigation allowed an accurate diagnosis of the patient’s lesion. Complementary testing of serum and/or CSF for autoantibodies will enlarge the diagnostic spectrum of neuropathological assessment and will allow a more precise diagnosis, avoid diagnostic delays, and permit the application of rapid immunomodulatory treatment. 

ADEM is a severe acute demyelinating disease with an annual incidence of 0.1 – 0.7 cases per 100.000 [1, 2]. It may occur spontaneously or follow infections and more often affects children than adults, with an average age of 5 – 8 years. Neuropathology is characterized by perivenous demyelination and inflammatory infiltrates that are dominated by T-cells, variable amounts of B-cells, and abundant macrophages and activated microglia [3]. Recent data suggest that ADEM represents a clinical syndrome that may be initiated by different pathogenetic triggers. Among children with an acute demyelinating syndrome about one third are seropositive for anti-MOG antibodies [4]. It is hypothesized that the disease is induced by a combination of a T-cell-mediated encephalitis with a demyelinating antibody response against a conformational epitope of MOG [5]. MOG positive ADEM differs from classical ADEM in respect to MR imaging and may have a higher risk to further develop into a relapsing or recurrent disease course [4, 6]. Histopathological findings may show abundant premyelinating oligodendrocytes and mild deposition of complement [7]. MOG antibodies are ideally detected by using live cell-based assays that present the native full-length human MOG protein [5]. The clinical and neuropathological diagnosis can be challenging and has important implications for treatment and prognosis. 

## Funding 

This work was supported by the Medical Scientific Fund of the Mayor of the City of Vienna, Project number 15022 and the Jubiläumsfonds der Österreichischen Nationalbank, Project number 16919. 

## Conflict of interest 

The authors report no conflict of interest. 

**Figure 1.**
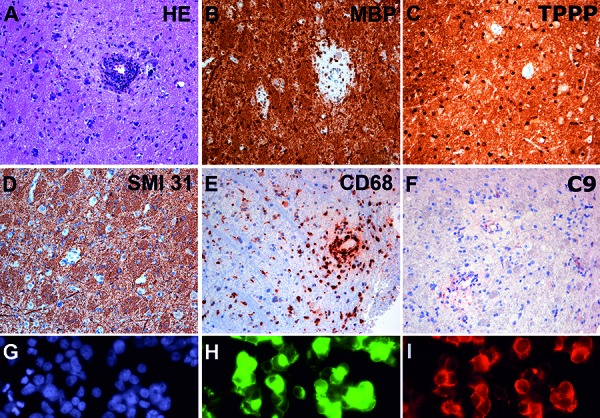
A: Hematoxylin & eosin (H & E) stained section shows CNS tissue with increased cellularity due to reactive astrocytes. A dense perivascular inflammatory infiltrate is observed in the center of the image. There is a slight perivascular pallor of the tissue. B: Immunohistochemistry for myelin basic protein (MBP) reveals prominent perivascular demyelination, while C: TPPP positive oligodendrocytes are well preserved. D: Immunohistochemistry for phosphorylated neurofilament SMI 31 of the same spot shows preservation of axons, confirming the selective demyelinating nature of the lesion. E: Immunohistochemistry for CD68 shows abundant activated microglia and macrophages that concentrate around the vessel. F: Mild perivascular complement deposits in the lesions (C9). G, H and I represent a cell-based assay for anti-MOG antibodies on HEK 293T cells. G (blue) represents nuclear staining, H (green) represents the effective transfection of the cells with the full-length MOG C-terminally fused with EGFP and I (red) shows the labeling of MOG-transfected HEK cells with the patient´s serum, confirming the presence of anti-MOG antibodies.
